# Piezoelectricity of hexagonal boron nitrides improves bone tissue generation as tested on osteoblasts

**DOI:** 10.3762/bjnano.16.78

**Published:** 2025-07-07

**Authors:** Sevin Adiguzel, Nilay Cicek, Zehra Cobandede, Feray B Misirlioglu, Hulya Yilmaz, Mustafa Culha

**Affiliations:** 1 Sabanci University Nanotechnology Research and Application Center (SUNUM), Istanbul, Turkey; 2 Chemistry and Biochemistry Department, College of Science and Mathematics, Augusta University, Augusta, GA, USA

**Keywords:** bone healing, hexagonal boron nitrides, human osteoblasts, nanomaterials, piezoelectricity

## Abstract

Bone tissue, also known as bone, is a hard and specialized connective tissue consisting of various bone cells. Internally, it has a honeycomb-like matrix providing rigidity to the bone and a piezoelectric feature contributing to bone remodeling. Bone remodeling is a crucial process involving osteoblastic replacement and resorption by osteoclastic cells to maintain structural integrity and mechanical properties of the bone tissue as it grows. However, in cases of fracture or degeneration, the natural self-regeneration process or inherent piezoelectricity of the body may not be sufficient to repair the damage. To address this, the use of piezoelectric nanomaterials (NMs) in bone tissue engineering was investigated. In this study, the influence of the piezoelectric hexagonal boron nitrides (hBNs) and barium titanate (BaTiO_3_) on human osteoblasts (HOb) was comparatively evaluated. The synthesized hBNs and purchased BaTiO_3_ were used after their full characterization by imaging and spectroscopic techniques. The piezoelectric behavior of both NMs was evaluated using piezoresponse force microscopy (PRFM). During in vitro studies, the piezoelectricity of the NMs was stimulated with ultrasound (US) exposure. The results showed that the NMs are not cytotoxic at the concentrations tested and the migration ability and calcium deposit formation of the cells treated with the NMs and upon US exposure were significantly increased. These results demonstrate that the hBNs have the potential to accelerate bone tissue regeneration and promote bone healing. These findings offer a promising avenue for developing new therapies for bone-related injuries and conditions requiring significant bone remodeling.

## Introduction

The skeletal system is a vital component of the human body, serving both structural and reservoir functions. It provides support, protection, and mobility while also storing essential minerals and lipids, regulating endocrine functions, and producing blood cells. Bone tissue undergoes continuous remodeling, where old bone is replaced with new tissue, a process that becomes particularly prominent once bones reach peak mass and continues through adulthood, replacing most of the skeleton approximately every decade [1]. Hydroxyapatite crystals (Ca_10_(PO_4_)_6_(OH)_2_) form through the nucleation of calcium and phosphate ions, imparting stiffness and resistance to bone. Additionally, collagen and noncollagenous matrix proteins contribute to bone formation by offering a scaffold for hydroxyapatite deposition [2–3].

Bone tissue consists of four main cell types: bone lining cells, osteoclasts, osteoblasts, and osteocytes, which coordinate bone resorption and deposition. Bone lining cells cover bone surfaces and play a key role in osteoclast differentiation [4–5]. Osteoclasts are large, multinucleated cells responsible for bone resorption through lysosomal enzymes and acid secretion [6]. Osteocytes, the longest-lived bone cells, arise from osteoblast differentiation and regulate bone maintenance [7]. Osteoblasts, found on bone surfaces, drive bone formation through two key steps: matrix deposition and mineralization. During matrix deposition, collagen and noncollagenous proteins are secreted, while proteoglycans help form the organic matrix [8]. During matrix mineralization, calcium and phosphate ions generate hydroxyapatite crystals within the matrix region. Once bone formation is complete, some osteoblasts become embedded within the matrix, transforming into osteocytes, which leads to structural changes [2]. Bone has an intrinsic regenerative capacity, enabling new bone formation after injury through the piezoelectric properties of its natural collagen structure. The piezoelectricity of bone tissue facilitates regeneration by converting mechanical stimuli into electrical signals. This capability arises from the noncentrosymmetric structure of collagen, which allows both direct and inverse piezoelectric effects [9]. Mechanically induced electrical stimulation activates cellular signaling pathways that enhance osteogenesis and promote osteogenic differentiation [10]. Specifically, activation of the calcium/calmodulin pathway leads to the expression of transforming growth factor-β (TGF-β), bone morphogenetic proteins (BMPs), and other regulatory factors which control extracellular matrix production, bone homeostasis, tissue repair, and mesenchymal stem cell (MSC) differentiation [10–13]. However, while bone remodeling via piezoelectricity supports regular healing, it is typically insufficient to repair severe bone injuries. Therefore, external stimulation is often required to aid and accelerate recovery. Conventional methods for bone repair, such as autografts and allografts, come with several disadvantages, including limited availability, donor site morbidity, and risk of immune rejection [14]. To address these challenges, new strategies are being developed, including synthetic substitutes, bioactive porous polymer/inorganic composites, scaffolds combined with bioactive molecules, biomimetic fibrous substitutes, biomaterials-based 3D cell-printing substitutes, and nanoscaffolds incorporating stem cells [15].

Piezoelectric materials are also under active investigation for their potential application in bone regeneration therapies [2,8,16]. These materials can stimulate electrically excitable cells without requiring an external power source, making them highly attractive for biomedical applications such as treating cardiac arrhythmias and promoting bone regeneration [17–18]. Mechanical stimulation, notably ultrasound (US), can induce a piezoelectric response in these materials [19]. In clinical practice, low-intensity pulsed ultrasound is commonly applied at intensity ranges between 20–50 mW/cm^2^ [20]. Combining piezoelectric materials including polymers such as polyvinylidene fluoride (PVDF) and inorganic materials such as barium titanate (BaTiO_3_) and hexagonal boron nitride (hBN) with ultrasound (US) stimulation is an emerging therapeutic approach. While polymers such as PVDF require electrical poling and often exhibit lower piezoelectric activity, inorganic materials inherently possess stronger piezoelectric properties and greater mechanical stability, making them particularly attractive for biomedical applications. [21–22]. Barium titanate (BaTiO_3_) is one of the most well-known piezoelectric nanomaterials (NMs), characterized by a cubic structure with four polymorphs which change depending on the temperature and a high dielectric constant. All its phases exhibit ferroelectric properties, except for the cubic crystalline phase [23]. The size and crystal structure of BaTiO_3_ can vary based on the synthesis method. Notably, BaTiO_3_ holds promise as a bone-like graft and as a nano–bio interface, thanks to its excellent cytocompatibility and its positive influence on cell proliferation driven by its piezoelectric characteristics [17,24]. It is particularly suitable for tissue engineering applications, especially in promoting osteogenesis [25]. Furthermore, BaTiO_3_ can be combined with other materials to create composite scaffolds with osteoconductive properties. Studies have demonstrated that BaTiO_3_ enhances osteogenesis and promotes osteocyte differentiation [26–27]. In this study, BaTiO_3_, a well-established piezoelectric material, was used as a benchmark for comparison with hexagonal boron nitride (hBN).

Hexagonal boron nitride (hBN) is a two-dimensional (2D) material, structurally similar to graphene, consisting of alternating boron and nitrogen atoms arranged in a hexagonal lattice. It is well known for its excellent thermal stability, electrical insulation, chemical inertness, and mechanical strength. Importantly, hBN exhibits piezoelectric properties due to its noncentrosymmetric crystal structure, making it a promising material for biomedical applications such as bone regeneration, where it can convert mechanical stimuli into bioelectrical signals. Additionally, the biocompatibility and ability of hBN to be integrated into composite scaffolds enhance its potential as a nano–bio interface in tissue engineering and regenerative medicine [28–29]. The crystalline structure of hexagonal boron nitride (hBN) consists of in-plane B–N bonds that are sp^2^ hybridized, polarized, and strongly covalent. Its most stable form is a hexagonal lattice of alternating boron and nitrogen atoms, with a bond length of ≈1.45 Å and an AA stacking arrangement held together by σ bonds. Between adjacent hBN layers, the B–N atoms are bound by weak van der Waals forces, contributing to a wide bandgap of 3.9–4.6 eV, influenced by significant electronegativity differences [30–31]. The piezoelectric property of 2D hBN arises from its noncentrosymmetric structure, supported by these van der Waals interactions [32]. The excellent biocompatibility of hBN makes it attractive for diverse applications, including cosmetics, drug delivery, cancer treatment, orthopedic implants, pharmaceutical lubricants, and tissue engineering [33]. In bone tissue engineering, hBN is valued for its stability and boron-containing compounds, which support bone regeneration, especially in fracture healing [34]. For example, Atila et al. (2016) developed an hBN–hydroxyapatite (HA) composite, demonstrating hBN as a promising implant material [35]. Al-Saadi et al. (2017) leveraged the mechanical stability of hBN to create hBN-impregnated silane bioimplants, which resisted human body fluid exposure for over 96 h [36].

Despite these advantages, the commercial use of hBN remains limited due to challenges such as low synthesis scalability, unavoidable defects, and inhomogeneity. Producing high-quality hBNs with controlled layer numbers depends heavily on precursor selection, ambient gas conditions, and substrate of choice [37]. Emerging solutions include both top-down (mechanical and chemical exfoliation) and bottom-up (chemical or physical atom assembly) synthesis methods, with chemical vapor deposition (CVD) standing out as a promising approach for large-area production [38].

This study aims to investigate novel, noninvasive stimulation of osteoblasts using commercial BaTiO_3_ and CVD-synthesized hBN. The materials were characterized using spectroscopic, imaging, and thermal techniques, followed by assessment of their piezoelectric properties via piezoresponse force microscopy (PRFM). In vitro studies on human osteoblasts (HOb) under ultrasound (US) exposure examined proliferation, cellular uptake, ROS levels, noncytotoxic dosing, and migration capacity. Additionally, osteoblastic differentiation and calcium deposition, as markers of end-stage osteoblast differentiation, were evaluated.

## Materials and Methods

### Synthesis and characterization of hexagonal boron nitrides

Hexagonal boron nitride was synthesized using boric acid (Sigma-Aldrich, Germany) and ammonia as the boron and nitrogen sources. Specifically, 1 gram of boric acid was mixed with 5 mL of 25% aqueous ammonia solution (Sigma-Aldrich, Germany) to create a suspension, which was then poured onto a silicon carbide (SiC) plate (Civelek Porselen, Turkey) and dried at 100 °C for 15 min. The plate was subsequently placed in a tubular furnace (Protherm, PTF 14/50/450) and heated to 1300 °C at a rate of 8 °C/min under an ammonia atmosphere for 2 h. After cooling to approximately 570 °C, the hBN material was gently scraped from the SiC surface and stored at room temperature. The synthesized hBN and commercial BaTiO_3_ nanoparticles (Sigma-Aldrich, Germany) were characterized using several analytical techniques. Transmission electron microscopy (TEM, JEOL ARM 200 CF, 200 keV) was employed to examine morphology and particle size by drop-casting 1 μL of hBN suspension (prepared in deionized water) onto a TEM grid. Fourier-transform infrared spectroscopy (FTIR, Shimadzu IRAffinity-1S) and Raman spectroscopy (Renishaw, 532 nm laser) were used to analyze chemical bonds and assess crystallinity. UV–visible spectroscopy (Varian Cary UV-Vis-NIR) measured optical properties across the 200–800 nm range, while dynamic light scattering (DLS, Malvern Panalytical) provided data on hydrodynamic size and zeta potential after dispersing 1 mg of material in 1 mL of deionized water and sonicating for 30 min. Finally, piezoresponse force microscopy (PFM, Nanomagnetic Instruments, UK) was applied to evaluate nanoscale piezoelectric behavior using self-aligned conducting EFM probes with a spring constant of 2.8 N/m and a resonance frequency of 75 kHz.

### In vitro analyses

To investigate the influence of hBNs and BaTiO_3_ with US therapy on osteoblast cell activity, the HOb cell line (cell line no. 406-05a, European Collection of Cell Cultures, UK) was chosen due to its sensitivity to mechanical and electric stimuli. Cell viability, reactive oxygen species (ROS) detection, cellular uptake, scratch, and von Kossa staining assays (Abcam, UK) were performed to investigate the main interactions between HOb cells and NMs with US stimulation. HOb cells were cultured in a High-Glucose Dulbecco’s Modified Eagle Medium/Nutrient Mixture F-12 (DMEM/F-12) (Pan Biotech) medium containing 10% fetal bovine serum (FBS) (Pan Biotech), 1% penicillin streptomycin (PS, Capricorn Scientific), and 1% ʟ-glutamine (Pan Biotech). Cells were incubated at 37 °C with 5% percent CO_2_ and 90% humidity. All experiments were performed after cells reached 100% confluence, and the NMs treatment procedure was the same. Both types of NMs were dispersed in cell culture medium using an ultrasonic bath before treatment. Using an ultrasonic bath, US was applied to cells after NMs treatment and within the incubation period, 3 times for 5 s daily at 37 kHz for all in vitro experiments. After the incubation, the medium was removed and washed with phosphate buffered saline (1X PBS, Pan Biotech) before measurements, and all reagents to be added were prepared in DMEM/F-12 medium. In all in vitro assays, only cells (control, C) and US-exposed cells (C+US) were used as control groups.

#### Cell viability assay

The cell viability assay was performed with the Alamar Blue (Biorad, US) colorimetric assay to determine the nontoxic concentration levels of both NMs and the impact of US exposure on viability. The oxidized form of the dye enters the cytosol when it is added to the cell culture, and it is converted to its reduced form by the mitochondrial enzyme activity of living cells [39]. Cells at a density of 10^4^ cells/well were seeded in 96-well plates for overnight incubation. Cells were treated with NMs with increasing concentration values (0.25, 0.5, 1, 5, and 10 µg/mL) and incubated for 24 h. Then, the Alamar Blue % solution was added to each well to incubate for 3 h at 37 °C and 5% CO_2_. Afterward, the absorbance was measured at 570–600 nm to determine cell viability via a Tecan microplate reader. The linear regression method calculated the half-maximal inhibitory concentration (IC50) values.

#### Reactive oxygen species detection assay

The ROS detection was carried out using the 2’,7’-dicfloroflurescin diacetate (DCFDA) (D6883-50MG; *M*_w_: 487.29 g/mol) probe, chosen specifically for its ability to detect the presence of cellular hydrogen peroxide (H_2_O_2_) in HOb cells following treatment with NMs and US exposure. First, the cells were seeded in 96-well plates at 10^4^ cells/well and incubated for 24 h. Then, the cells were treated with NMs with increasing concentrations (0.25, 0.5, 1, 5, and 10 µg/mL) and incubated for 24 h. Then, the DCFDA solution was prepared with a final concentration of 10 µM/mL and given to each well in a 100 µL volume. Each plate was coated with aluminum foil and incubated for 45 min. Afterward, fluorescence and absorbance were measured at the 485/535 nm range by a microplate reader (Tecan, Switzerland).

#### Cellular uptake assay

According to the logic of the NMs uptake procedure by flow cytometry, an increasing percentage of side scatter (SSC%) shift indicates growth in granulation, so that the cellular uptake of NMs and the effect of US exposure on uptake can be determined. HOb cells were seeded in 24-well plates at 5 × 10^4^ cells/well and incubated for 24 h. Then, cells were treated with NMs with increasing concentration values (1, 5, and 10 µg/mL) and left for incubation for 24 h. Then, the cells were harvested and centrifuged, and cell pellets were suspended with 200 µL PBS for cellular uptake analysis with flow cytometry (BD LSR Fortessa Flow Cytometer, US) and the FlowJo software was used for interpretation of data.

#### Scratch assay

A scratch assay was performed to investigate the migration ability of HOb cells exposed to US and NMs under reduced serum culture conditions to inhibit cell proliferation [40]. Firstly, HOb cells were seeded in 12-well plates at 10^5^ cells/well and incubated for 24 h. Then, the cells adhered to the wells were vertically scratched using a 200 µL pipet tip, and then washed with PBS to remove the unattached cells. Afterward, the cells were treated with NMs, suspended in serum-free media with increasing concentration values (1, 5, and 10 µg/mL), and left for incubation for 24 h. Images of the cells were taken at 0, 6, and 24 h using an inverted light microscope to observe the scratch closure (cellular migration). These images were analyzed using the Wound Healing Image Analysis Platform (Wimasis) and the ImageJ software, and the percent scratch closure was calculated.

#### Von Kossa staining assay

To estimate the influence of NMs and US stimulation on osteoblastic differentiation, calcium depositions as an osteoblastic end differentiation marker was detected via the von Kossa staining procedure. Coloring is based on the conversion of calcium salts into silver salts. The calcium ions bound to the phosphates are replaced by the silver ions in the silver nitrate solution used in the staining procedure [41]. HOb cells were seeded in 24-well plates at 6 × 10^3^ cells/well and incubated for 24 h. After 24 h, cells were treated with NMs with increasing concentration values (1, 5, and 10 µg/mL). After 1, 4, and 7 days of treatment of cells with NMs, 5% silver nitrate solution was added to each well and exposed to UV light for 50 min. Afterward, the cells were washed with diH_2_O three times, and 5% sodium thiosulfate solution was added to each well to stop the reaction. After 3 min, the cells were rinsed under running tap water for 1 min and in diH_2_O for 2 min. Then, the cells were incubated with the Nuclear Fast Red Solution for 5 min and rinsed with running tap water and diH_2_O two times. After washing, the wells were rapidly dehydrated with absolute ethanol (Sigma-Aldrich, Germany) by changing them three times. The images of each well were taken using an inverted microscope (Carl Zeiss, Germany) to observe calcium deposition formation.

#### Cell viability assay for von Kossa staining assay

The cell viability assay was performed with Alamar Blue colorimetric assay. Cells at a density of 6 × 10^3^ cells/well were seeded in 24-well plates. After 24 h, the cells were treated with NMs with increasing concentration (1, 5, and 10 µg/mL) and incubated for 1, 4, and 7 days. Then, 10% Alamar Blue solution in cell culture medium was prepared and added to each well to incubate for 3 h. Afterward, the absorbance of the solution was measured at 570–600 nm via a microplate reader (Tecan, Switzerland) to determine cell viability.

## Results and Discussion

### Characterization of hexagonal boron nitrides and BaTiO_3_

While hBNs were synthesized with a CVD method in house, BaTiO_3_ with an average size of 50 nm was purchased. Both were thoroughly characterized using a variety of techniques including FTIR, Raman, UV–vis, DLS, and PRFM to confirm their physicochemical properties.

#### Characterization of hexagonal boron nitrides

**Figure 1:**
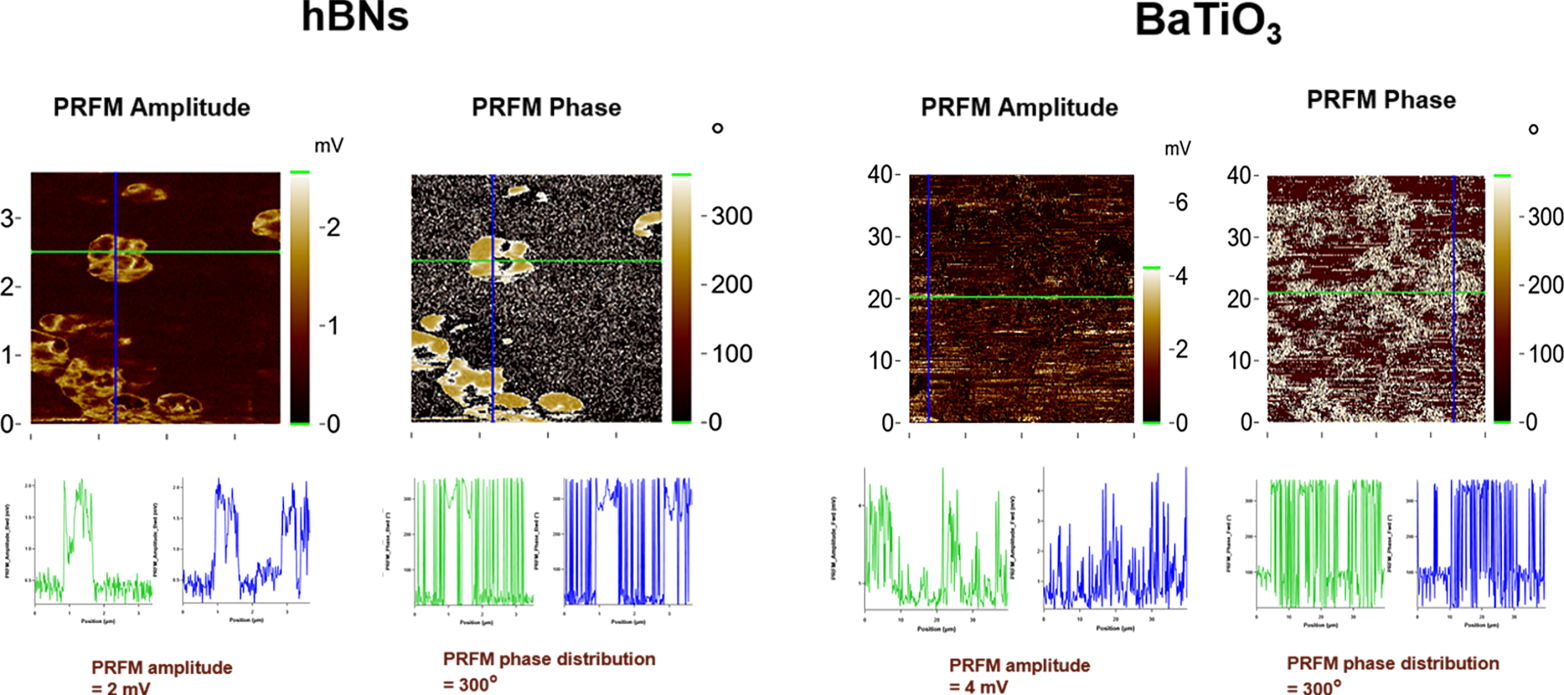
PRFM amplitude and phase images of hBNs and BaTiO_3_.

The TEM images of the synthesized hBNs are shown in Supporting Information File 1, Figure S3 (a) and (b). As seen, hBNs have platelet-like morphology with varying sizes between 50–100 nm, consistent with the literature [42]. Three characteristic peaks of hBNs on the FTIR spectrum in Supporting Information File 1, Figure S3 (c) appeared at 1363, 785, and 661 cm^−1^, attributed to the in-plane B–N stretching vibration, B–N bonding, and B–N–B out-of-plane bending vibration, respectively [43]. The broad band at 3200 cm^−1^ could be attributed to stretching vibrations of hydroxyl groups (O–H) of hBNs or humidity. Supporting Information File 1, Figure S3 (d) displays the Raman spectrum of hBNs, which reveals a dominant peak at 1367 cm^−1^, in line with the B–N vibrational mode (E_2g_) as consistent with the literature [44–45]. Due to its highly transparent properties, hBN can transmit 99% of light in the 250–900 nm region. The UV–vis absorption peak at 203 nm is shown in Supporting Information File 1, Figure S3 (e), corresponding to its optical bandgap energy [46]. Supporting Information File 1, Figure S3 (f) illustrates that the hydrodynamic size of hBNs in aqueous suspension is approximately 120 nm. The zeta potential value is −42.5 ± 1.18 mV, indicating greater negativity than −30 mV, demonstrating stability [47]. Figure 1 shows PRFM amplitude and PRFM phase images of hBNs. The amplitude image presents the strength of hBNs piezoelectric response, which was recorded as 2 mV. This acquired amplitude response measures the effective piezoelectric coefficient, which can be correlated with the polarization magnitude. The observed PRFM amplitude contrast showed that grains have different piezo responses. According to the applied electric field, bright areas correspond to regions with active piezoelectric response, while dark regions indicate piezoelectric activities in the opposite direction of the applied electric field on the surface [48]. In the PRFM images, green horizontal lines represent lateral (in-plane) piezoresponse, while blue vertical lines correspond to vertical (out-of-plane) piezoresponse. These signals reflect the directional electromechanical behavior of the material and are not scanning artifacts. According to the applied electric field, the acquired phase signals determine the polarization direction of hBNs [49]. The direction and angle of polarization of piezoelectric hBNs were recorded by phase contrast measurement, and it was found to be 300°. This high value indicates that hBNs gave spontaneous responses as polarization to external stimulation.

#### Characterization of BaTiO_3_

The FTIR spectrum of BaTiO_3_ is shown in Supporting Information File 1, Figure S4 (a). A characteristic peak of BaTiO_3_ was observed at 1420 cm^−1^, corresponding to crystalline Ba–Ti–O [50]. Additionally, slight peaks appeared at 1630 and 3480 cm^−1^ attributed to OH-stretching and OH-deformation vibrations due to moisture content, respectively [51]. The Raman spectrum, displayed in Supporting Information File 1, Figure S4 (b), revealed the presence of Raman active vibration modes of BaTiO_3_ in its tetragonal phase, indicating its polycrystalline nature [51–52]. The peaks at 244, 307, 513, and 715 cm^−1^ correspond to the Raman shift of BaTiO_3_. Intense peaks appeared at 244 and 307 cm^−1^, accounting for the vibration in the TiO_6_ group. The peak that appeared at 513 cm^−1^ is caused by the vibration that resulted from oxygen atom displacement [53]. The peak around 185 and 244 cm^−1^ corresponds to A1(TO). A peak at around 307 cm^−1^ is a specific peak of the tetragonal BaTiO_3_ phase, indicating B1, E(TO+LO). In the case of the cubic phase, the peak that appeared around 305 cm^−1^ is decreased [53]. The peak that appeared at 513 cm^−1^ indicates [A1(TO), E(TO)] and the peak that appeared at 715 cm^−1^ correspond to [A1(LO), E(LO)] [54]. The UV–vis spectrum presented in Supporting Information File 1, Figure S4 (c) showed an absorption edge increase of BaTiO_3_ at around 300 nm [55]. Supporting Information File 1, Figure S4 (d) shows that the DLS spectrum provides the hydrodynamic size and zeta potential values of BaTiO_3_ as 44 ± 6.68 nm and −22.74 ± 1.67 mV, respectively. This zeta potential value indicates the potential for particle aggregation. Additionally, the amplitude and phase images in Figure 1 indicated a polarization magnitude of 4 mV, which was higher than the piezoelectric response of hBNs, as expected due to the perovskite ceramic structure of BaTiO_3_ [56]. The phase contrast image also revealed a polarization magnitude of 300°, indicating the presence of spontaneous electrical polarization in BaTiO_3_ after stimulation.

### In vitro cell interaction studies

#### Cell viability assay

**Figure 2:**
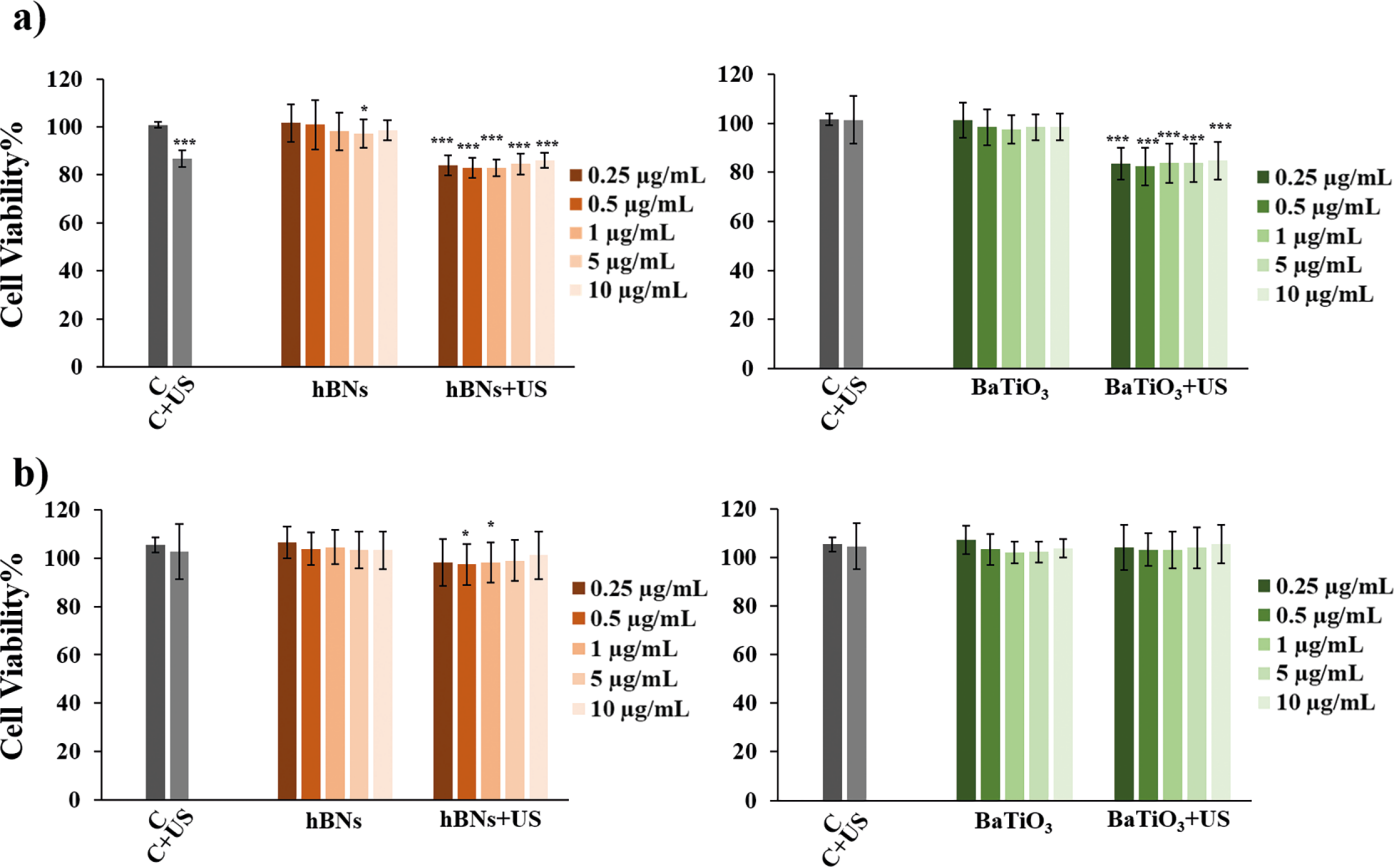
Viability of HOb cells treated with hBNs, hBNs+US, BaTiO_3_, and BaTiO_3_+US for (a) 24 h and (b) 48 h. Statistically significant change compared to the control group were indicated with asterisk (Student’s *t*-test, (*p ≤ 0.05, ***p ≤ 0.001).

The concentrations and exposure-time-dependent cell viability data for the cells treated with hBNs, hBNs+US, BaTiO_3_, and BaTiO_3_+US are shown in Figure 2. As observed, none of the tested concentrations of the NMs exhibited cytotoxicity on HOb cells over a 48 h period. However, the viability of cells treated with hBNs+US for 24 h was decreased to 80% for all concentrations of hBNs. Both hBNs and BaTiO_3_ showed the same behavior with the US exposure for 24 h. This reduction in cell viability can be attributed to the transient mechanical effects by ultrasound exposure. Although applying US to the environment is a noninvasive mechanical stimulation, it may still cause acoustic streaming, acoustic microstreaming, and cavitation. This mechanical effect may cause fluid flow in the extracellular environment, creating temporary membrane deformation and tension on osteoblasts [57]. Moreover, sonoporation, a mechanical effect of US, may lead to a temporarily perturbation of cell membrane homeostasis and disruption of the cell membrane [58]. Additionally, US exposure could cause cells to detach from the surface, potentially leading to their removal during the washing process with PBS before adding the Alamar Blue reagent to the well [59]. However, no significant cytotoxicity was observed after 48 h, suggesting that cells recovered from the transient mechanical stress and restored their membrane function and viability over time. The use of low-intensity ultrasound (20–50 mW/cm^2^) in this study supports the noninvasive nature of the stimulation and minimizes the likelihood of permanent cellular damage.

#### ROS detection assay

**Figure 3:**
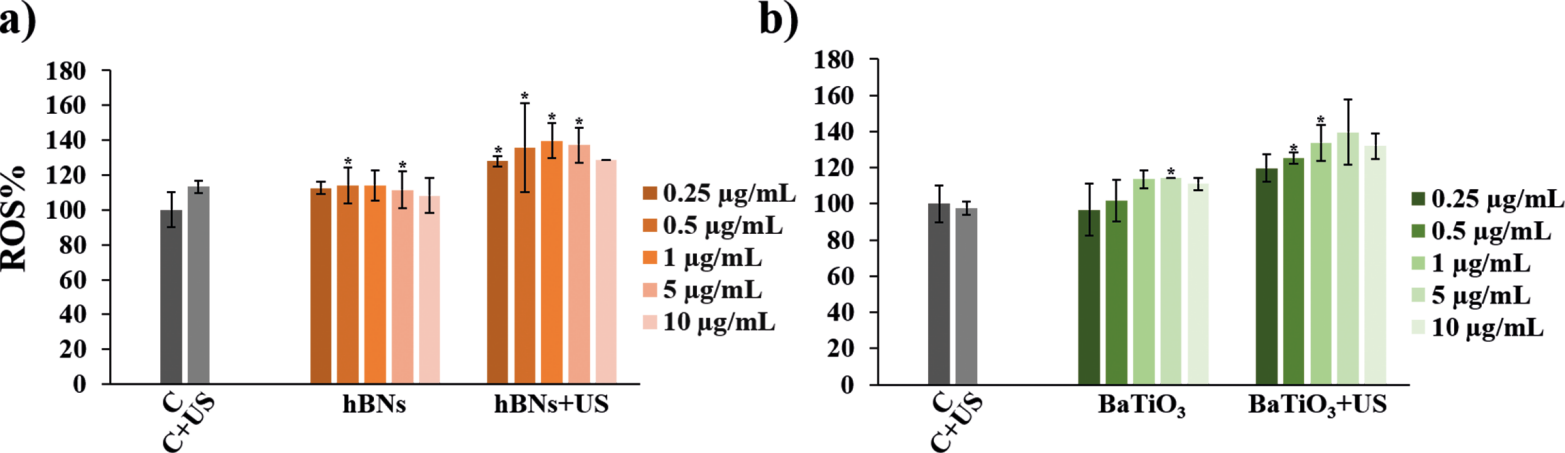
Revealed ROS% level of HOb cells treated with a) hBNs and hBNs+US, b) BaTiO_3_ and BaTiO_3_+US. Statistically significant changes compared to the control group were indicated with asterisks (Student’s *t*-test, *p ≤ 0.05).

Concentration-dependent ROS% levels in the cells treated with hBNs, hBNs+US, BaTiO_3_, and BaTiO_3_+US are shown in Figure 3. In the control+US group used for hBNs-treated HOb cells, the ROS% level increased to 113%. Conversely, there was no increase in the ROS% in the control+US group for BaTiO_3_. Regardless of concentration, the ROS% level increased by 20% in the cells treated with hBNs+US and BaTiO_3_+US compared to those treated with only hBNs and BaTiO_3_ alone. This difference can be attributed to the polarization of hBNs, which generates an electric current and initiates an interfacial redox reaction that generates ROS [60]. As a result, the ROS% levels in the cells treated with hBNs+US and BaTiO_3_+US were very close. Following the application of US, a significant increase in ROS levels was observed. Specifically, it has been demonstrated that US enhances ROS production via NADPH oxidases [61–62], which subsequently activates MAPK/ERK signaling pathways which promote osteoblast differentiation [61]. This explains how increased intracellular ROS levels can contribute to the enhancement of calcium deposits in osteoblasts. Additionally, it has been emphasized that ROS, when produced at physiological levels, serves as a crucial signaling molecule that supports osteogenic differentiation and mineralization [63].

IC50 values, which represent the concentration inhibiting a biological response by 50% [64], were calculated using linear regression. The IC50 values of hBNs and BaTiO_3_ were found to be 53 and 85 μg/mL, respectively. Based on these values, the highest concentrations that could achieve the maximum effect were selected. This approach ensures that the treatments are both effective and minimize potential adverse effects, providing a balanced assessment of the impact of US and NMs treatments on cellular responses. Based on the study results regarding cell viability and ROS% levels, 1, 5, and 10 µg/mL were chosen for further experiments to reflect a strategy to explore dose-response relationships in a nontoxic range.

#### Cellular uptake assay

**Figure 4:**
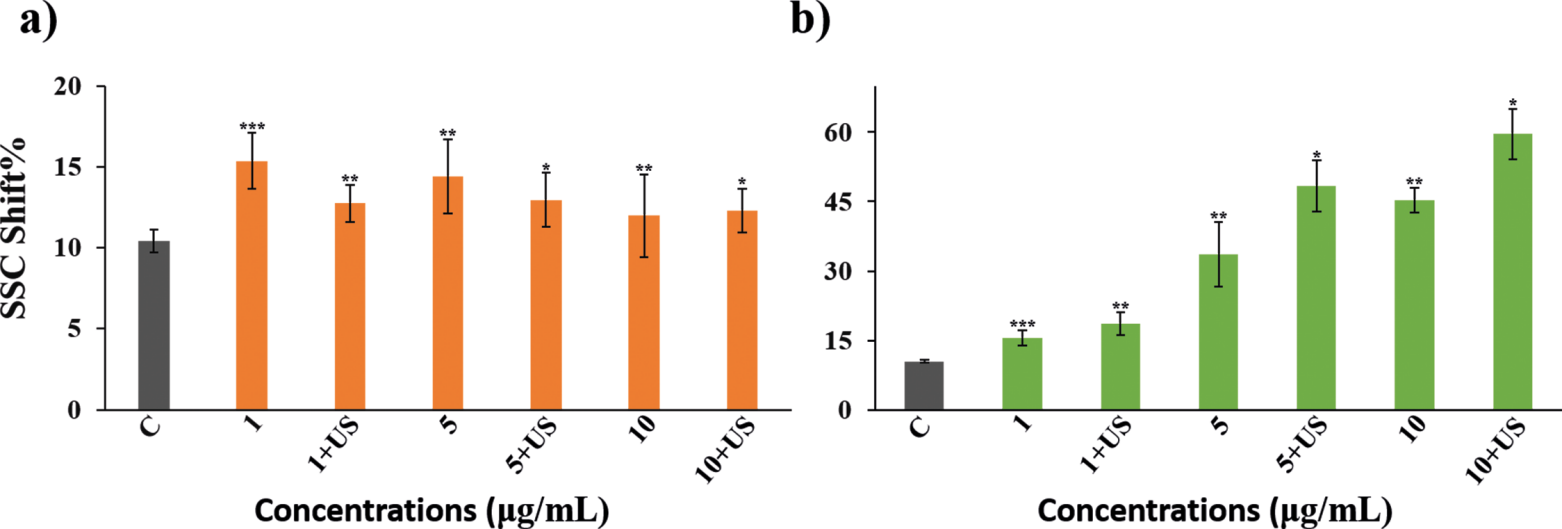
SSC shift % results of HOb cells treated with a) hBNs and hBNs+US, b) BaTiO_3_ and BaTiO_3_+US. Statistically significant changes compared to the control group were indicated with asterisks (Student’s *t*-test, (*p ≤ 0.05, **p ≤ 0.01, and ***p ≤ 0.001).

Figure 4 displays the SSC% shift observed for both NMs compared to the control group. The SSC% shift in hBNs and hBNs+US treated cells remained consistent across concentrations and were nearly identical. Despite their different structures, sizes, charges, and shapes, which affect their internalization [65], BaTiO_3_ was taken up by cells in a concentration-dependent manner, leading to increased cell granulation, as depicted in Figure 4. Furthermore, the SSC% shift in BaTiO_3_+US treated cells significantly increased with concentration compared to those treated with BaTiO_3_. This can be attributed to the smaller size and hydrophilic nature of BaTiO_3_, which facilitates easier cellular uptake through the cell membrane compared to that of hBNs. The observed increase in SSC% shift under NMs+US treatment suggests that US exposure may enhance cellular uptake mechanisms. This enhancement can be attributed to US exposure generating stress and increasing alterations in membrane fluidity, thereby facilitating higher uptake of NMs, as illustrated in Figure 4 [66].

#### Scratch assay

**Figure 5:**
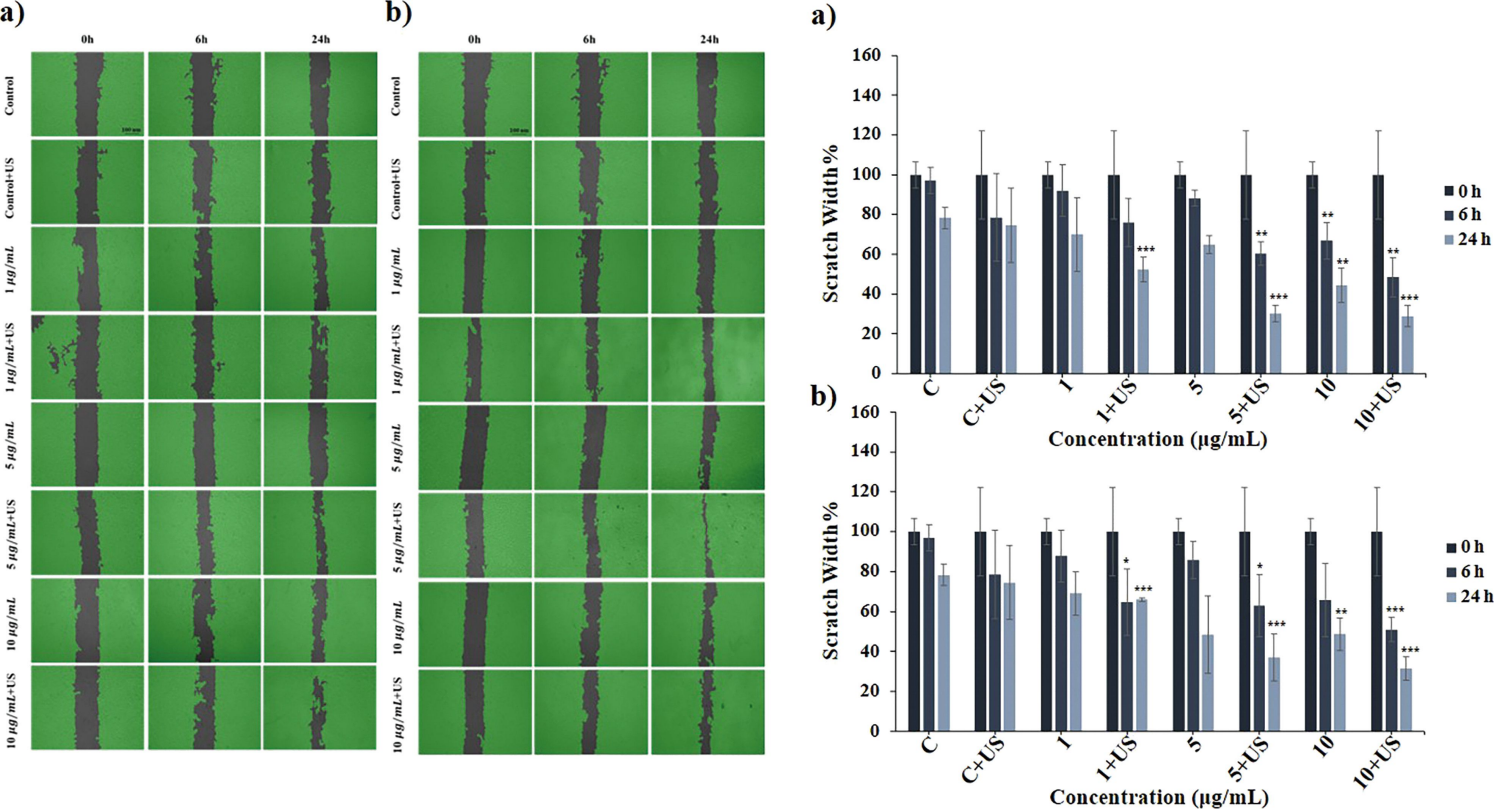
Scratch images and scratch width graphs of in vitro scratch assays applied to HOb cells at 0, 6, and 24 h after treatment with a) hBNs and hBNs+US and b) BaTiO_3_ and BaTiO_3_+US. The scratch width was calculated by ImageJ. Statistically significant changes compared to the control group were indicated with asterisks (Student’s *t*-test, (*p ≤ 0.05, **p ≤ 0.01, and ***p ≤ 0.001).

A scratch assay was conducted to evaluate the effects of the NMs and the US exposure on the migration ability of HOb cells. A pipette tip was used to create a scratch in the monolayer of cells, and the time required to close the scratch under US exposure was assessed. The results of the assay are presented in Figure 5. The scratch width in the control+US groups was narrower than in the control group, suggesting that US exposure may enhance the natural migration ability of cells at 6 h, a significant decrease in scratch width was observed in cells treated with hBNs (10 µg/mL) and hBNs+US (5 and 10 µg/mL). By 24 h, the scratch width had decreased significantly across all tested concentrations, with the most dramatic effects seen in the cells treated with hBNs (5 and 10 µg/mL) and hBNs+US (10 µg/mL). For cells treated with BaTiO_3_ (1 µg/mL) and BaTiO_3_+US (1 µg/mL), the scratch width was similar at 24 h. However, at varying concentrations, a significant decrease in scratch width was observed at 24 h. The scratch width in cells treated with BaTiO_3_ (5 and 10 μg/mL) was similar, but cells treated with BaTiO_3_+US (10 μg/mL) showed a more pronounced effect on migration ability than those treated with BaTiO_3_+US (5 μg/mL) at 24 h. Overall, the scratch width was significantly reduced in cells treated with hBNs+US (5 and 10 µg/mL) and BaTiO_3_+US (5 and 10 µg/mL). However, cells treated with hBNs+US exhibited a significantly greater scratch width reduction than those treated with BaTiO_3_+US. The enhanced migration ability can be attributed to the biological activity of boron released from hBNs, potentially originating from boric acid, a degradation product of hBNs [67–68]. Although hBNs are chemically stable under neutral conditions, Şen et al. have shown that hBNs synthesized from boric acid slowly degraded in oxidative and hydrolytic environments, releasing boron over time. This boron is present in physiological media mostly in the form of boric acid which is the thermodynamically stable and biologically active species at pH ≈7.4 [68–69]. The literature indicates that hBN-based nanostructures can enhance bone recovery and osteointegration [35,70–71]. Additionally, the WNT/β-catenin and BMP signaling pathways have been well documented in the literature for their roles in promoting osteoblast migration and differentiation, particularly in response to mechanical stimuli such as US exposure [72–74]. These pathways are crucial for cellular processes that are essential for bone repair and regeneration, which could explain the superior performance of hBNs in promoting osteoblast migration compared to that of BaTiO_3_ under US exposure. This suggests that the combination of hBNs and US may provide a more favorable environment for activating these pathways, leading to enhanced osteoblast function and bone healing.

#### Von Kossa staining

**Figure 6:**
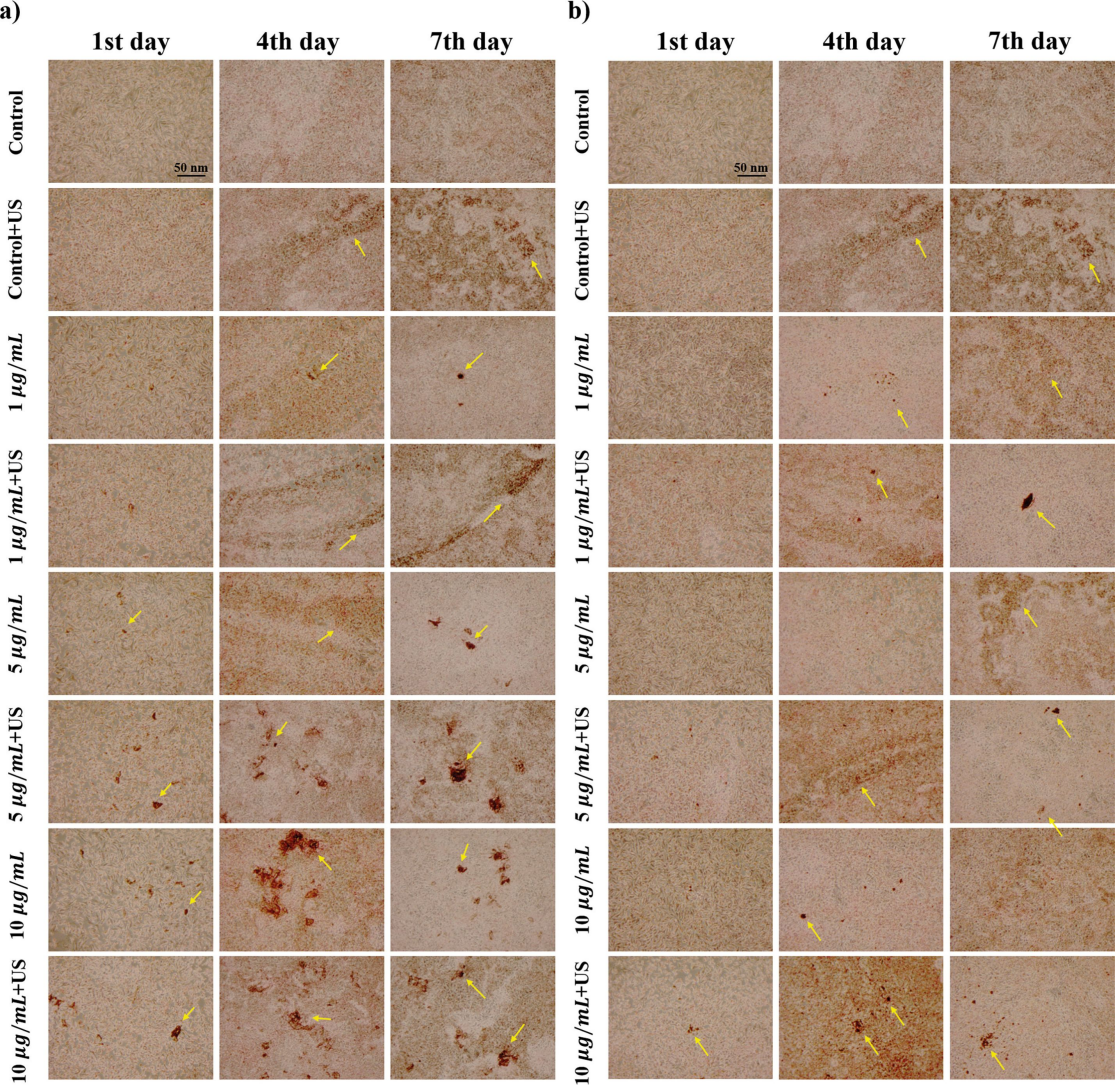
Images of von Kossa staining applied to HOb cells treated with a) hBNs and hBNs+US and b) BaTiO_3_ and BaTiO_3_+US. Scale bar = 50 nm.

**Figure 7:**
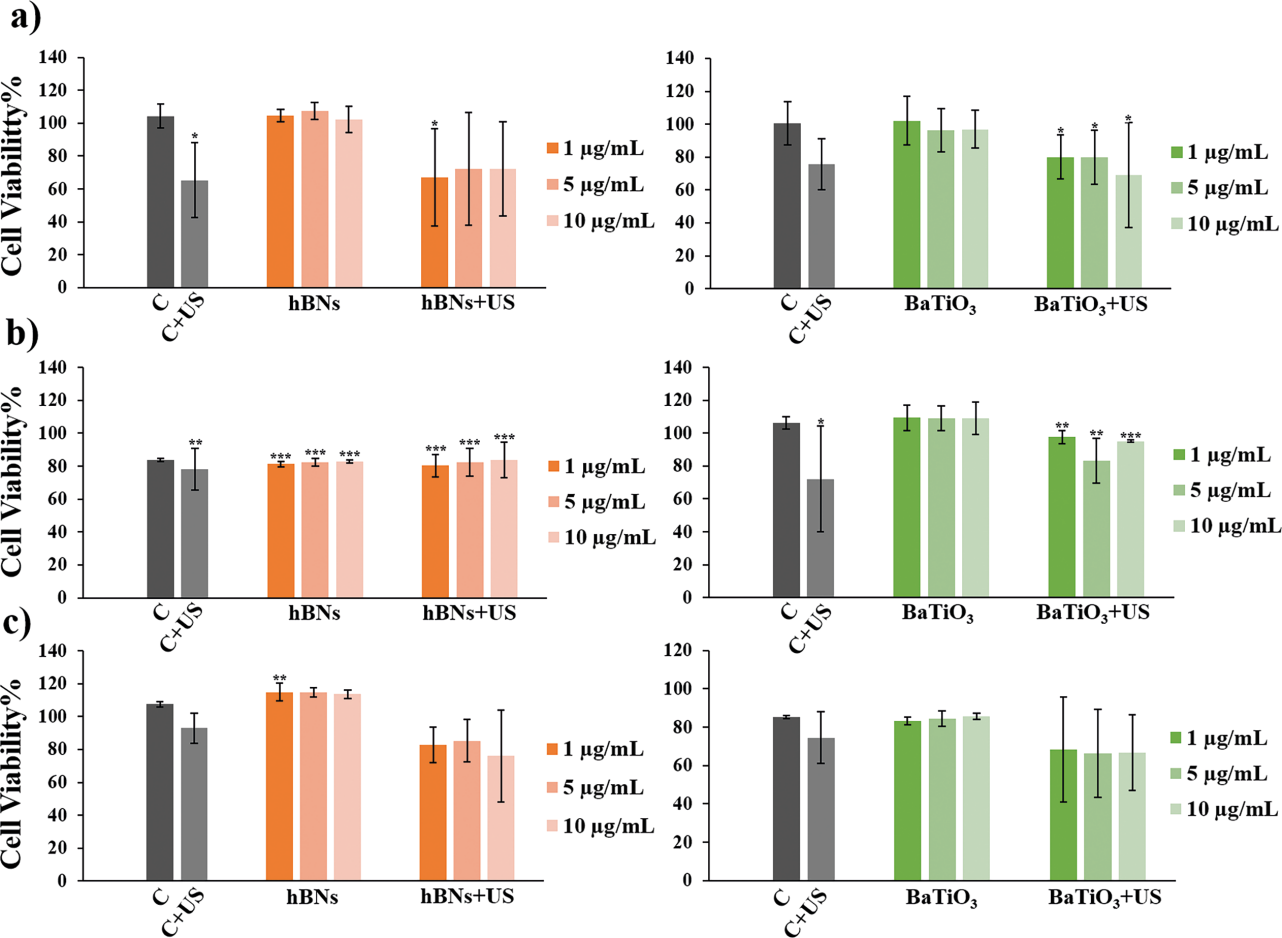
Cell viability results of HOb cells treated with hBNs, hBNs+US, BaTiO_3_, and BaTiO_3_+US for (a) 1st day, (b) 4th day, and (c) 7th day. Statistically significant change compared to the control group were indicated with asterisk (Student’s *t*-test, (*p ≤ 0.05, **p ≤ 0.01, and ***p ≤ 0.001).

Figure 6 shows the von Kossa staining images of HOb cells treated with hBNs and hBNs+US. The dark brown spots and suspended brown stains indicate calcium deposition, serving as a marker for osteoblast end differentiation [75–76]. In the control+US group, calcium depositions increased on the 1st, 4th, and 7th days compared to that of the control group. This increase is expected due to the activation of calcium ion channels by US exposure, leading to enhanced Ca^2+^ uptake [77]. Cells treated with hBNs+US (5 and 10 µg/mL) exhibited higher calcium deposition than those treated with hBNs alone. Similarly, cells treated with BaTiO_3_+US showed higher calcium deposition than those treated with BaTiO_3_ alone. This increase in calcium deposition can be explained by US-induced ROS production through mechanotransduction, which targets TRPV4 calcium channels, leading to an influx of calcium into the cells [61–62]. Additionally, the activation of MAPK/ERK signaling pathways by ROS can promote osteoblast differentiation and the formation of calcium deposits [61–62]. This process is supported by literature as a mechanism in which ROS plays a critical role in the mineralization process in osteoblasts [63]. In addition, the slow degradation of hBNs, resulting in the release of boric acid, a boron compound that supports the osteogenic response, may further contribute to the increase in calcium deposition by inducing intracellular signaling pathways [65]. However, calcium depositions decreased on the 7th day in cells treated with BaTiO_3_+US (5 and 10 µg/mL), as shown in Figure 6. A seven-day Alamar Blue cell viability test, shown in Figure 7c, was conducted to investigate this decrease. It revealed that the viability of cells treated with BaTiO_3_+US was lower than that of cells treated with BaTiO_3_ alone. By the 7th day, the reduced cell viability in the treated wells can be attributed to the lack of space for further proliferation and expansion, leading to cell detachment from the surface.

## Conclusion

In this study, we comparatively investigated the effects of commercially available BaTiO_3_, a well-known ferroelectric and piezoelectric material, and hBNs, successfully synthesized via the CVD method and recognized for their piezoelectric properties on osteoblast cell activity in the presence and absence of US exposure. The results demonstrate that both NMs exhibited robust piezoelectric properties and confirmed their exceptional biocompatibility, with no toxicity observed even at the highest concentrations tested. The mechanical stress induced by the US prompts micromechanical interaction with cells. This, in turn, triggers signaling pathways that profoundly influence cellular behavior. Our findings showed that US application significantly increased ROS levels, which in turn may have promoted osteoblast differentiation and enhanced calcium deposition through pathways such as MAPK/ERK signaling. Von Kossa staining revealed that hBNs, when combined with US, induced greater calcium deposition in osteoblasts compared to that of BaTiO_3_, suggesting a stronger osteogenic response. This effect could be attributed to the mechanotransduction effects of US, possibly influencing calcium influx and related cellular processes. Additionally, the slow degradation of hBNs, releasing boric acid – a compound known to support osteogenesis – further contributed to the increase in calcium deposition by interacting with cellular pathways [78–79]. The study also highlighted the potential of hBNs to significantly enhance cell migration under US exposure, as observed in the scratch assay. This enhanced migration may be linked to the unique piezoelectric properties of hBNs, which could interact with cellular mechanisms that improve cell motility. Interestingly, despite the lower cellular uptake of hBNs compared to that of BaTiO_3_, hBNs were more effective in promoting cell migration and osteogenic differentiation when combined with US. The involvement of pathways such as WNT/β-catenin and BMP in these processes, as suggested in the literature, might explain the enhanced osteogenic responses observed [71–73]. These findings indicate the broader implications of hBNs in applications such as wireless tissue stimulation and noninvasive therapeutic strategies, positioning them as promising candidates in advanced medical treatments aimed at enhancing bone regeneration and healing. In conclusion, our groundbreaking findings demonstrate the superior osteogenic potential of hBNs over BaTiO_3_ when exposed to US, driven by their unique piezoelectric properties and the beneficial effects of boric acid [78] on cellular processes [77–78]. These natural piezoelectric NMs, particularly hBNs, not only hold significant potential for biocompatible wireless tissue stimulation applications but also envision a future where these materials could revolutionize noninvasive therapeutic strategies. The biocompatibility and effectiveness of hBNs make them a promising candidate for future therapeutic applications aimed at enhancing tissue regeneration and bone healing, potentially ushering in a new era of medical advancements.

## Supporting Information

This file includes schematic illustrations of hBN synthesis and in vitro experimental setup, along with supplementary data such as TEM images, FTIR and Raman spectra, UV–vis results, DLS analysis, and topographic images of hBNs and BaTiO_3_ nanoparticles.

File 1 Additional figures.

## Data Availability

Data generated and analyzed during this study is available from the corresponding author upon reasonable request.
