# Supplementary material for: Piezoelectricity of hexagonal boron nitrides improves bone tissue generation as tested on osteoblasts
Source: Beilstein J Nanotechnol. 2025 Jul 7;16:1068–81. doi: 10.3762/bjnano.16.78 (PMC12256782; doi:10.3762/bjnano.16.78)
Supplement: File 1 — Additional figures. [file Beilstein_J_Nanotechnol-16-1068-s001.pdf]

BEILSTEIN JOURNAL OF NANOTECHNOLOGY

Supporting Information

for

Piezoelectricity of hexagonal boron nitrides improves bone tissue generation as tested on osteoblasts

Sevin Adiguzel, Nilay Cicek, Zehra Cobandede, Feray B. Misirlioglu, Hulya Yilmaz and Mustafa Culha

Beilstein J. Nanotechnol. **2025,** *16,* 1068–1081. doi:10.3762/bjnano.16.78

Additional figures

License and Terms: This is a supporting information file under the terms of the Creative Commons Attribution License (https://creativecommons.org/licenses/by/4.0). Please note that the reuse, redistribution and reproduction in particular requires that the author(s) and source are credited and that individual graphics may be subject to special legal provisions.
The license is subject to the *Beilstein Journal of Nanotechnology* terms and conditions: (https://www.beilstein-journals.org/bjnano/terms)

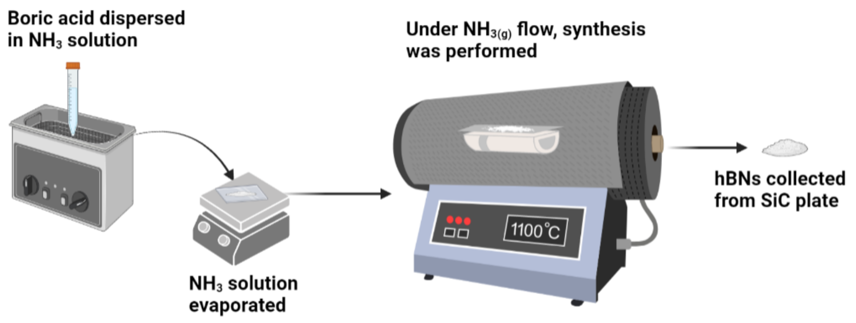

Figure S1: Schematic representation of the synthesis process of hBNs using the CVD method. Created in BioRender. Adıgüzel, Ş. (2025) https://BioRender.com/1xs905s. This content is not subject to CC BY 4.0.

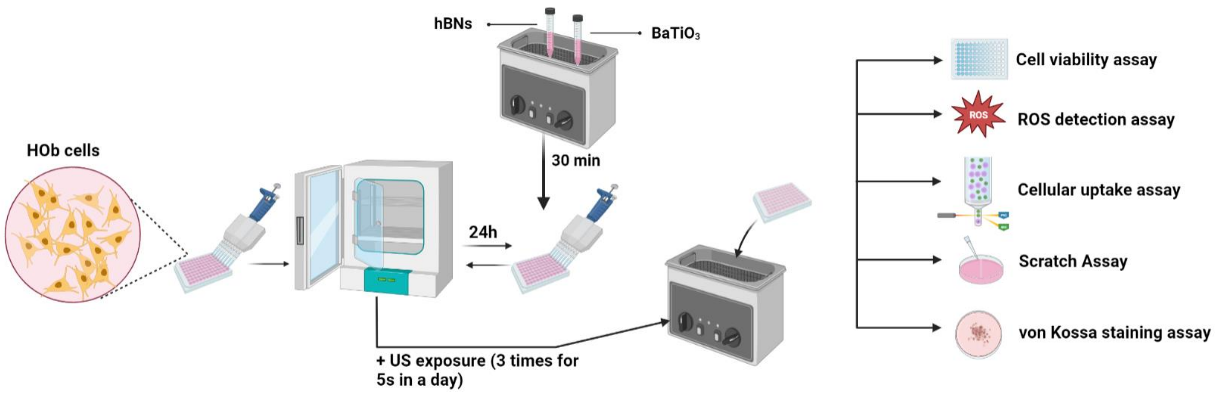

Figure S2: Schematic representation of the in vitro studies Created in BioRender. Adıgüzel, Ş. (2025) https://BioRender.com/4y6g1p7. This content is not subject to CC BY 4.0.

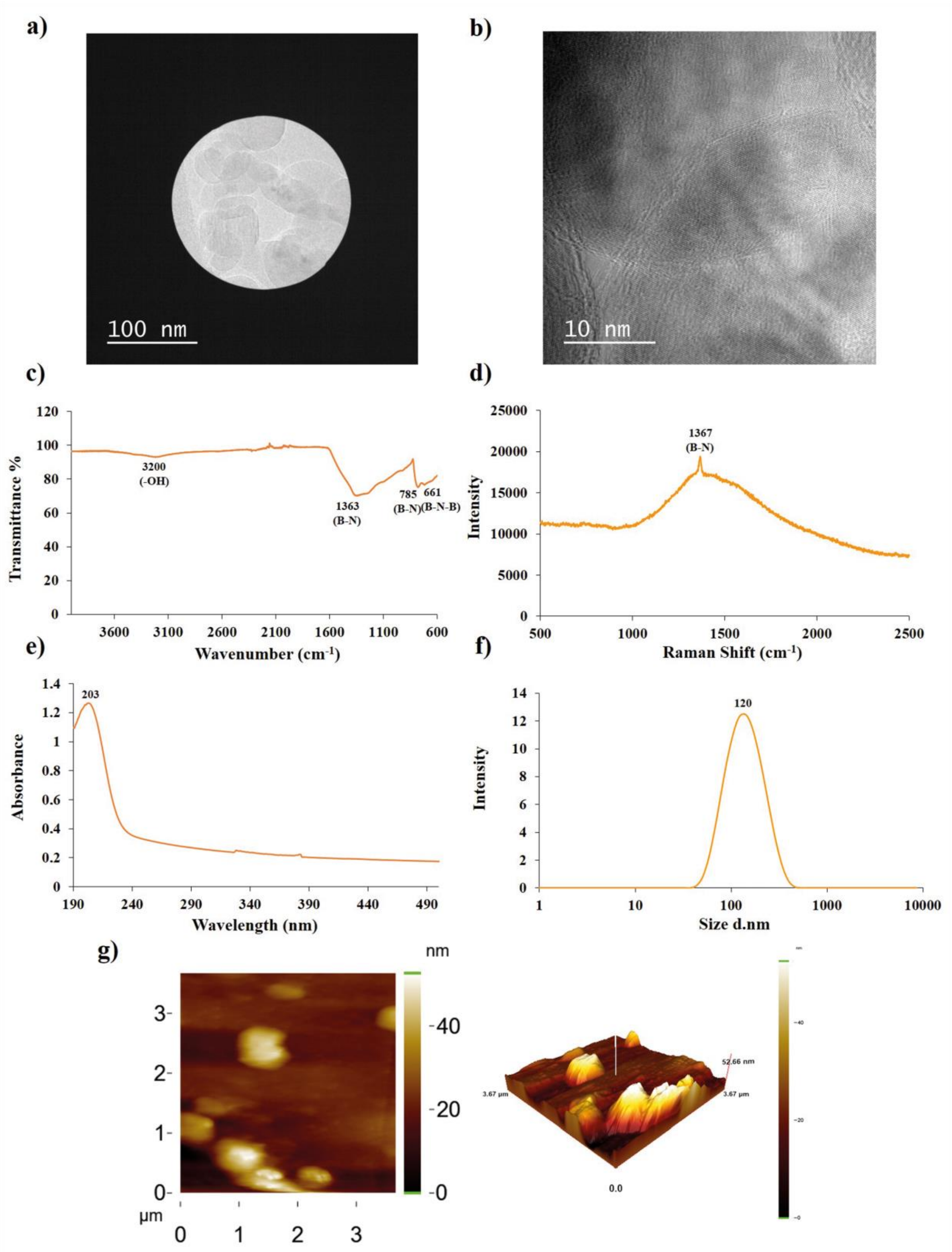

Figure S3: (a) and (b) TEM images with increasing magnifications, (c) FTIR, (d) Raman, (e) UV–vis, (f) DLS spectra, and (g) topography of hBNs.

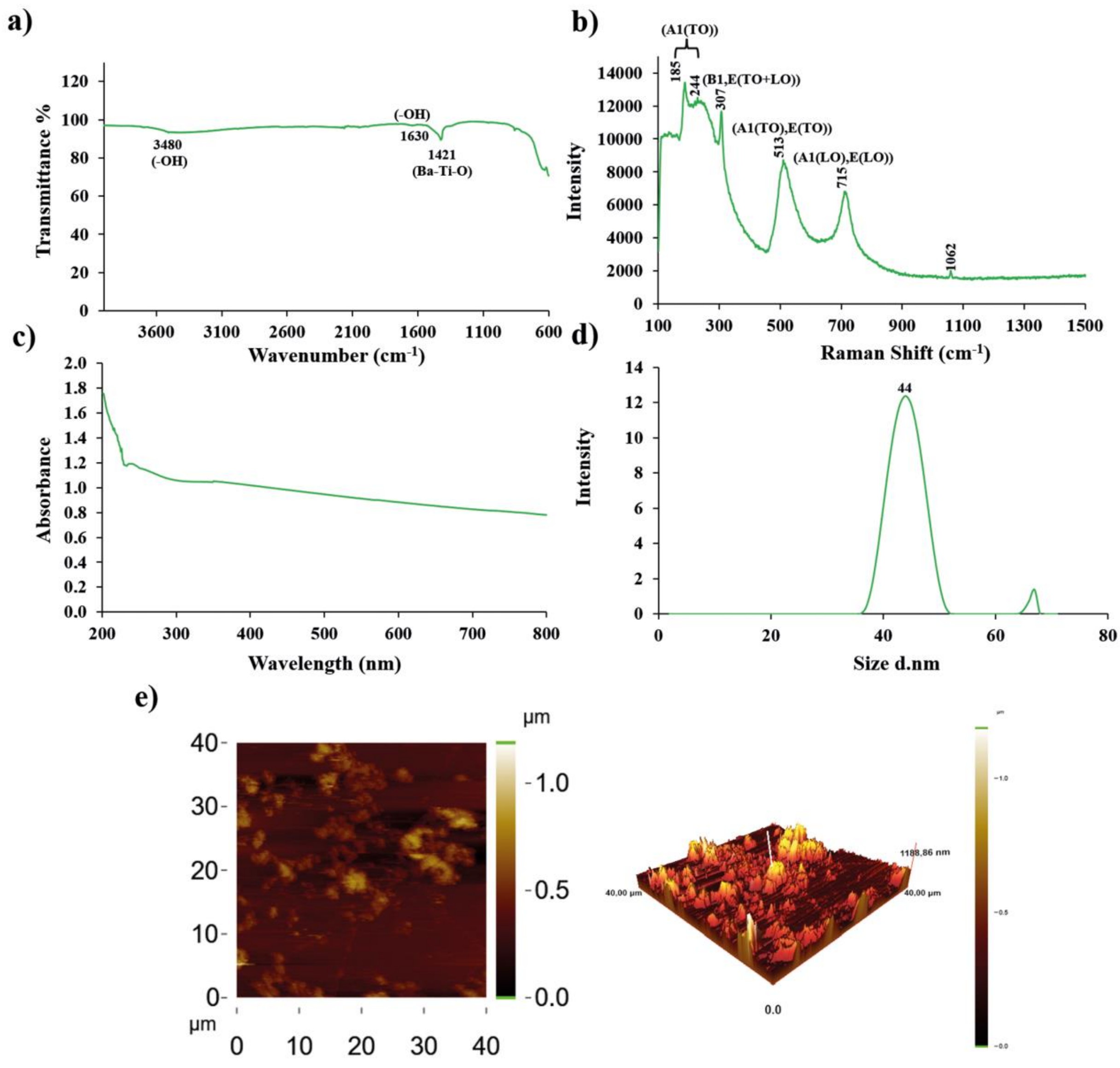

Figure S4: (a) FTIR, (b) Raman, (c) UV–vis, (d) DLS spectra, and (e) topography of $BaTiO_3$.
